# Synthesis and characterization of sulfonated poly(eugenol-co-allyleugenol) membranes for proton exchange membrane fuel cells

**DOI:** 10.1016/j.heliyon.2022.e12401

**Published:** 2022-12-17

**Authors:** Ngadiwiyana, Gunawan, Nor B.A. Prasetya, Tutuk D. Kusworo, Heru Susanto

**Affiliations:** a Chemistry Departement, Faculty of Science and Mathematics, Diponegoro University, Semarang, Central Java, Indonesia; b Chemical Engineering Departement, Faculty of Engineering, Diponegoro University, Semarang, Central Java, Indonesia

**Keywords:** Allyleugenol, Copolymer, Eugenol, Fuel cell, Sulfonation

## Abstract

The research of sulfonated eugenol-allyleugenol copolymer (SPEAE) based membrane for fuel cell from eugenol derivate had been conducted. First, eugenol was reacted with various weights of allyl eugenol to form eugenol-allyleugenol copolymer (PEAE). Determination of the optimum composition of PEAE was done by testing the swelling properties. Then, PEAE was sulfonated using concentrated sulfuric acid with time variations of 1, 2, 3, 4, and 5 h to form SPEAE. The SPEAE produced was tested for the degree of sulfonation, water uptake, cation exchange capacity, and membrane proton conductivity. In addition, the characteristics of the PEAE and SPEAE copolymer membranes were also analyzed using FTIR spectrophotometers, 1H-NMR, TGA, and DSC. The results showed that the copolymerization of eugenol:allyleugenol (EG:AEG) with a ratio of 10:1 gave the lowest swelling degree. The best SPEAE copolymer was obtained from sulfonation for 2 h with yield, degree of sulfonation, water absorption value, proton conductivity, and cation exchange capacity of 90.6%, 12.87%, 50.7%, 1.83 × 10^−5^ S cm^−1^ and 0.356 meq/g, respectively. FTIR analysis shows the formation of PEAE with the loss of the vinyl eugenol groups used to form the polymer and shows the formation of SPEAE in the presence of sulfonate groups from the sulfonation reaction. ^1^H-NMR also confirmed the presence of the PEAE and SPEAE copolymers. In addition, analysis of thermal properties with TGA and DSC also showed that sulfonate treatment could improve membrane stability.

## 1 Introduction

The development of polymers in the industry provides many opportunities to produce new materials that are useful in various fields such as drug delivery, anti-fungal [1], antibacterial and antioxidant [2], epoxy resin [3], composite materials [4], solar cells with active polymers [5, 6], sensor [7, 8] as well as electrochromic devices such as polymers for fuel cell membranes [9, 10]. Most synthetic polymers are made from a non-renewable resource (petroleum). The scarcity of petroleum deposits and the increasing negative impact on the environment underlies the development of novel polymers from biological materials. This is in line with the result of a study in the USA which estimates that by 2090, organic chemical products made from renewable natural resources will reach 90% [11].

Research to make new materials, in this case, polymers with renewable raw materials has begun to develop. Several studies to make polymer from renewable materials are modifying natural polymers such as cellulose [12, 13], starch [14], chitosan [15, 16], sodium alginate [17, 18] and pectin [19, 20]. The limited types and properties of natural polymers that exist, therefore, make the development of research for synthesizing polymers from renewable natural materials as a substitute for petroleum very interesting [2, 21].

One of the easily available renewable resources for polymer synthesis is eugenol. Eugenol is a phenolic compound that is the main component of clove oil (*Eugenia caryophyllata*) (70–90%) which can be easily extracted by adding base to form eugenol salts [22, 23]. Besides having a phenolic group (-OH), eugenol also has methoxy and allyl. The presence of an allyl group (-CH_2_CH = CH_2_) in eugenol allows polymerization to occur through a cationic addition polymerization reaction. Research on the synthesis of polymers from eugenol has been widely carried out and applied, such as for antibacterial and antioxidant polymers [2], and thermosetting renewable materials [24, 25].

The structure of eugenol polymer consists of aromatic rings substituted by hydroxyl (phenolic) and methoxy groups. The acidity of the phenolic group allows the process of releasing protons (H+) which have the potential to be used as proton exchange polymer membranes in fuel cells. This is consistent with several research developments on new polymers as fuel cell materials, particularly nonfluorinated polymers such as sulfonated polystyrene, sulfonated polyimide, and sulfonated poly (aryl ether sulfone) [26]. Müller et al., [27] succeeded in synthesizing PS/SEBS membranes from polystyrene (PS) and high-impact polystyrene (HIPS) doped poly(styrene-ethylene-butylene) (SEBS). Research shows the effect of sulfonation, where a membrane with a degree of sulfonation of 13% has a proton conductivity of 6.3 × 10^−9^ S cm^−1^, while HIPS/SEBS of a degree of sulfonation of 11% has a proton conductivity of 1.6 × 10^−9^ S cm^−1^ [27]. Ngadiwiyana et al. [28] performed sulfonation of polystyrene from the isolation of styrofoam waste and obtained conductivity value of 4.23 × 10^−8^ S cm^−1^.

Modification of polymeric materials can be carried out to meet the fuel cell membrane character specifications such as swelling properties, water uptake values, proton transfer capacity, and proton conductivity [27]. Swelling degree limitation of polyeugenol can be done by copolymerization of eugenol with crosslinking agents such as allyl eugenol which can be synthesized from eugenol [29]. Eugenol is very possible to be modified since it has two allyl groups. Additional allyl groups are carried out by reacting eugenol with allyl bromide [21, 29, 30, 31]. This allyl group can act as a polymerization agent through addition polymerization. The presence of two allyl groups in allyl eugenol is expected to be crosslinked/branched in resulting eugenol-allyleugenol copolymer (PEAE). The number of monomers in the copolymerization process determines the structure of the polymer formed and the properties of the polymer [32].

Increasing the conductivity and cation exchange capacity (CEC) of the resulted polymer can be done by sulfonation of PEAE using sulfuric acid [33]. This sulfonation reaction follows the electrophilic aromatic substitution reaction. Sulfonate groups are super acids that can act as proton transfers [9, 34, 35, 36, 37], so this stage becomes very important for the development of materials for fuel cell polymer membranes. Therefore, this work aims to synthesize PEAE from eugenol, followed by sulfonation using sulfuric acid to obtain sulfonated PEAE (SPEAE). Parameters of SPEAE related to the ability as a fuel cell membrane, including proton conductivity, water uptake, and swelling properties were studied.

## 2 Experiment

### 2.1 Materials and instrumentation

The materials used were eugenol, BF_3_O(C_2_H_5_)_2_ catalyst, diethyl ether, ethanol, chloroform, sodium hydroxide, sodium chloride, anhydrous sodium sulfate, universal pH, phenolphthalein indicator from Merck, while sulfuric acid from Mallincrokdt AR 98% and allyl eugenol (99%) synthesized by our research group. The equipment and instruments used in the study included a set of glassware, ovens, burettes, Ubbelohde viscometer, FTIR PerkinElmer Spotlight 200 S/N 96766–96681, ^1^H-NMR (JEOL 500 Hz, JNM ECA 500), LCR Meter (HOKI 3522-50 LCR HITESTER), Shimadzu DSC-60, and Thermogravimetry Analyzer (TG7300).

### 2.2 Synthesis of PEAE

Allyl eugenol with weight variations of 1, 5, 10, 15, and 20% was put into a three-neck flask and eugenol (10.64 mg) was added. The polymerization reaction was carried out by adding 2 mL of BF_3_O(C_2_H_5_)_2_ at room temperature and under a nitrogen atmosphere. After the mixture thickened, the polymerization was stopped by adding methanol (2 mL). The mixture was then dissolved in diethyl ether and washed with distilled water until the pH became neutral. After washing, the organic phase was dried using anhydrous Na_2_SO_4_, while the solvent was evaporated to produce a PEAE membrane.

### 2.3 Synthesis of SPEAE

The SPEAE synthesis process was carried out by mixing 10 g of PEAE and 250 mL of chloroform. The mixture was then stirred until it became homogeneous and 25 mL of concentrated sulfuric acid was added at room temperature. After that, the solution was heated at 65 °C with varying sulfonation reaction times for 1, 2, 3, 4, and 5 h. After the sulfonation reaction was completed, the reaction flask was cooled below 10 °C with a refrigerator to stop the sulfonation reaction and produce a polymer solution slowly. The polymer solution was then immersed in cold distilled water to form two layers which continued with separation. Finally, the results of the separation were washed with distilled water until the pH became neutral and dried in an oven at 60 °C.

### 2.4 Characterization

#### 2.4.1 Proton nuclear magnetic resonance (1H NMR) analysis

PEAE and SPEAE membranes were analyzed with the JEOL 500 Hz spectrometer instrument JNM ECA 500 with the resonant frequency set at 400 MHz. As much as 2–5% by weight of the polymer solution was prepared in the main solvents in the form of dimethyl sulfoxide-d6 (DMSO-d6) and tetramethylsilane (TMS) as internal standards. The analysis was carried out by setting the acquisition parameters at a spectral window of 6000 Hz (15 ppm), 2-second acquisition time, 1-second relaxation delay, and 55° pulse angle. Scans were performed 64 times at a controlled temperature of 30 °C.

#### 2.4.2 Fourier-transform infrared spectroscopy

The synthesized membranes were analyzed using FT-IR spectrophotometer (Perkin Elmer Spotlight 200 S/N 96766–96681) to determine the functional groups in the membrane composition. FT-IR spectra were recorded at room temperature with wave numbers of 400–4000 cm^−1^ in a KBr matrix.

#### 2.4.3 Thermal properties analysis

Thermal analysis of the membrane was carried out by thermogravimetric (TGA) and differential scanning calorimetry (DSC) tests to investigate the thermal degradation process, thermal properties, and glass transition temperature. The TGA test was carried out with a Thermogravimetry Analyzer (TG7300) instrument with a heating rate of 5 °C min^−1^ under nitrogen flow and platinum as reference material at a temperature range of 50–800 °C. Meanwhile, differential scanning calorimetry (DSC) was tested with a Shimadzu DSC-60 calorimeter equipped with a cooling system. Measurements were made at a temperature range of 50–530 °C with a scanning speed of 5 °C min^−1^. Experiments were carried out in a stream of nitrogen gas, and glass transition temperature (T_g_) values were obtained from the second heating scan thermogram.

#### 2.4.4 Swelling properties and surface area change

Determination of swelling properties is determined by the change in membrane thickness (%). To ensure the difference in thickness values arising from swelling, measurements of changes in the surface area of the membrane were also carried out. Measurements were made on allyl eugenol samples 1, 5, 10, 15, and 20% with 3 repetitions to obtain accurate results. The process began with heating in a vacuum oven at 70 °C for 1 day to measure the thickness (t_dry_) and surface area (A_dry_) of the dry membrane at various points. Then the membrane was soaked for 1 day in 2 mL of distilled water. After the time had elapsed, the membrane was removed from the bath and wiped with a soft tissue to absorb excess water. Re-measurement was carried out at various points as in the initial measurement. Changes in surface area and swelling properties of the membrane were determined using Eqs. (1) and (2), respectively [38, 39].

(1) $$Change\;of\;Surface\;Area\;\left(\%\right)=\frac{A_{wet}-A_{dry}\;}{A_{dry}}\times 100\%$$

(2) $$Change\;of\;Thickness\;\left(\%\right)=\frac{t_{wet}-t_{dry}\;}{t_{dry}}\times 100\%$$

where A and t are the surface area and thickness of the membrane, respectively.

#### 2.4.5 Water uptake

The SPEAE membrane was dried in a vacuum oven at 70 °C for 1 day and then weighed to obtain the SPEAE dry weight (W_dry_). Dried SPEAE was then soaked in water for 1 day at room temperature. After the time was up, the membrane was taken out and the excess water on the surface of the membrane was removed using a soft tissue. The wet weight of the SPEAE membrane (W_wet_) was then determined and the water uptake (WU) was calculated using Eq. (3) [38].

(3) $$WU\;\left(\%\right)=\frac{W_{wet}-W_{dry}\;}{W_{dry}}\times 100\%$$

#### 2.4.6 Cation exchange capacity and sulfonation degree

The cation exchange capacity (CEC) of the SPEAE membrane was determined by the acid-base titration method to determine the amount of H+ cations released from the membrane. 0.1 g of SPEAE membrane was weighed and then immersed in 10 mL of 0.1 M NaCl solution for 2 days. After that, the solution was filtered and the filtrate was titrated with 0.02 M NaOH and used phenolphthalein as an indicator [33]. By measuring the amount of NaOH consumed in the titration, the CEC value can be determined by Eq. (4). Meanwhile, the degree of sulfonation can then be determined from the CEC value obtained as in Eq. (5).

(4) $$CEC=\frac{Volume\;of\;NaOH\;consumed\;in\;titration\;x\;molar\;concentration\;of\;NaOH}{Weight\;of\;sample}$$

(5) $$DS=\frac{M_{P}\;x\;CEC}{1000-\left(M_{SO3H}x\;CEC\right)\;}$$

Where M_p_ is the molecular weight (g mmol^−1^) of the nonfunctional polymer (PEAE) repeat unit.

#### 2.4.7 Proton conductivity

The SPEAE membrane was pelleted to determine thickness using an Enerpac press, with a pressure and diameter of 300 Psi and 10 mm, respectively. Measurements were made with a frequency parameter of 100 kHz and a voltage of 20 mV under the same measurement conditions (25 °C and humidity at 50% RH). SPEAE pellets are conditioned by being clamped between 2 electrodes connected to the positive and negative poles of the LCR Meter (HOKI 3522-50 LCR HITESTER) to measure impedance. Area resistance measurements for each membrane were repeated 3 times to obtain accurate average data. The proton conductivity is then calculated using Eq. (6).

(6) $$\rho =\frac{R\times A}{I}\sigma =\frac{1}{\rho }$$

Where ρ is the resistivity (Ω.cm), A is the surface area of the sample (cm^2^), R is the resistance (Ω), I is the thickness of the sample (cm) and σ is the conductivity value (S cm^−1^ or Ω^−1^ cm).

## 3 Results and discussion

### 3.1 Synthesis of PEAE

**Figure 1:**
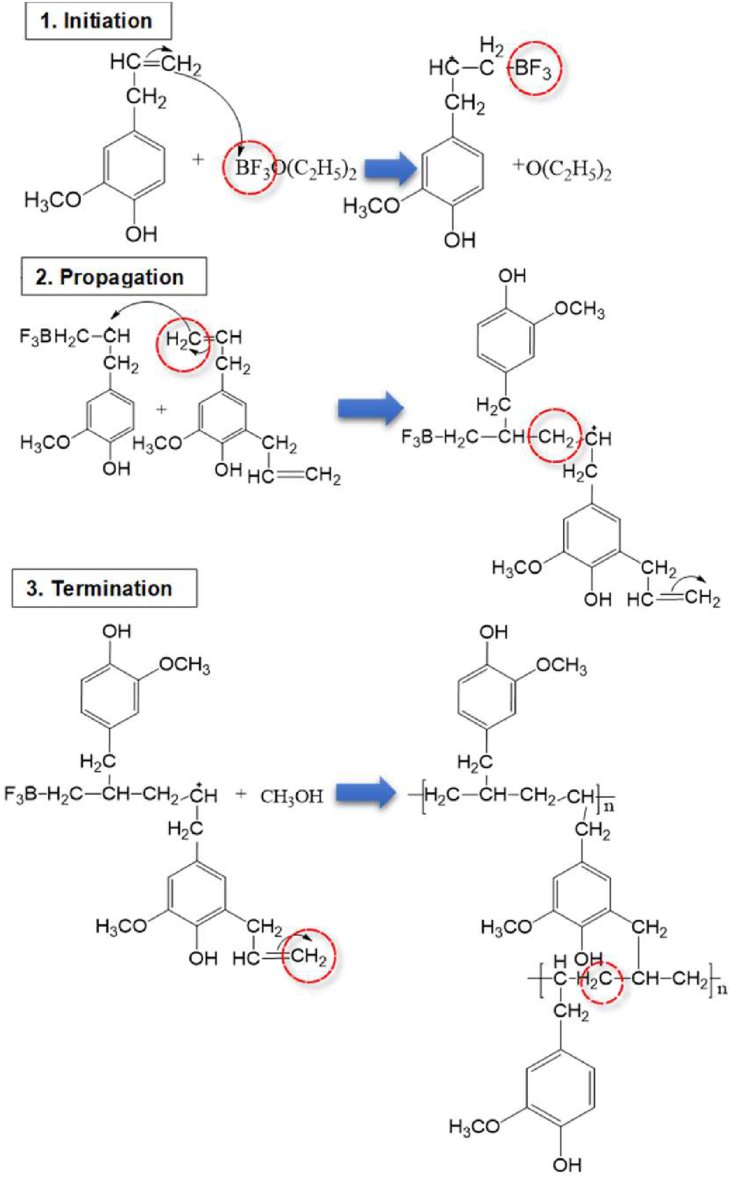
Copolymerization reaction in PEAE synthesis.

PEAE membranes were obtained by cationic addition polymerization reactions. This reaction goes through three stages: Initiation, propagation, and termination stages as shown in Figure 1.

At the initiation stage, BF_3_ will react with the eugenol or allyl eugenol monomer to produce a carbonium ion, which is characterized by the change in color of the solution to reddish brown. The next stage is called the copolymer propagation stage. At this stage the process of copolymer formation takes place. The last step is the termination, in which at this stage an amount of methanol is added which aims to stop the copolymerization reaction. The synthesis results in a brown PEAE copolymer with yields of around 90%.

**Figure 2:**
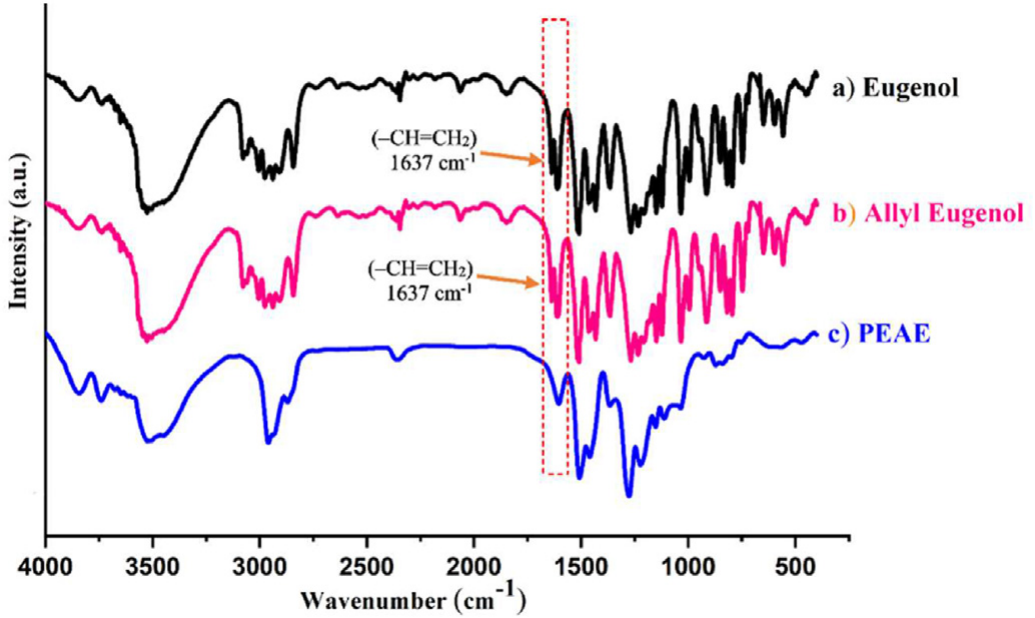
FTIR spectra (a) eugenol, (b) allyl eugenol, and (c) PEAE.

**Figure 3:**
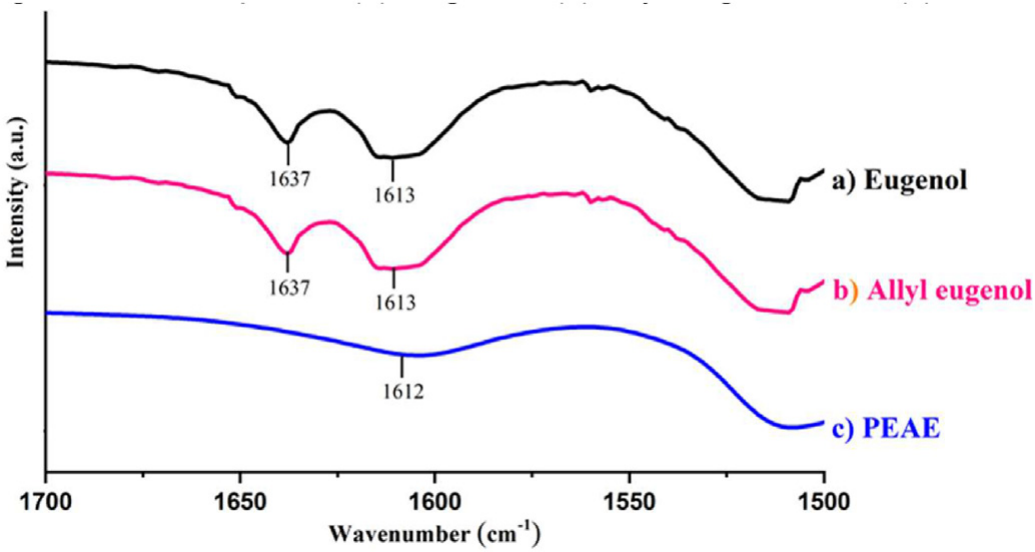
Magnification of the FTIR spectra at wavenumbers 1500–1700 cm^−1^ (a) eugenol, (b) allyl eugenol, and (c) PEAE.

The success of PEAE synthesis is proven by functional group analysis using FTIR as shown in Figure 2. The spectrum of PEAE has a clear difference with eugenol and allyleugenol as starting compounds. It is clarified by magnification in Figure 3 that the wave number area of 1500–1700 cm^−1^ shows the loss of vinyl group absorption (–CH=CH_2_) at a wave number of 1637 cm^−1^. This indicates that PEAE is formed due to the loss of the vinyl group used to form polymers such as the reaction listed in Figure 1.

**Figure 4:**
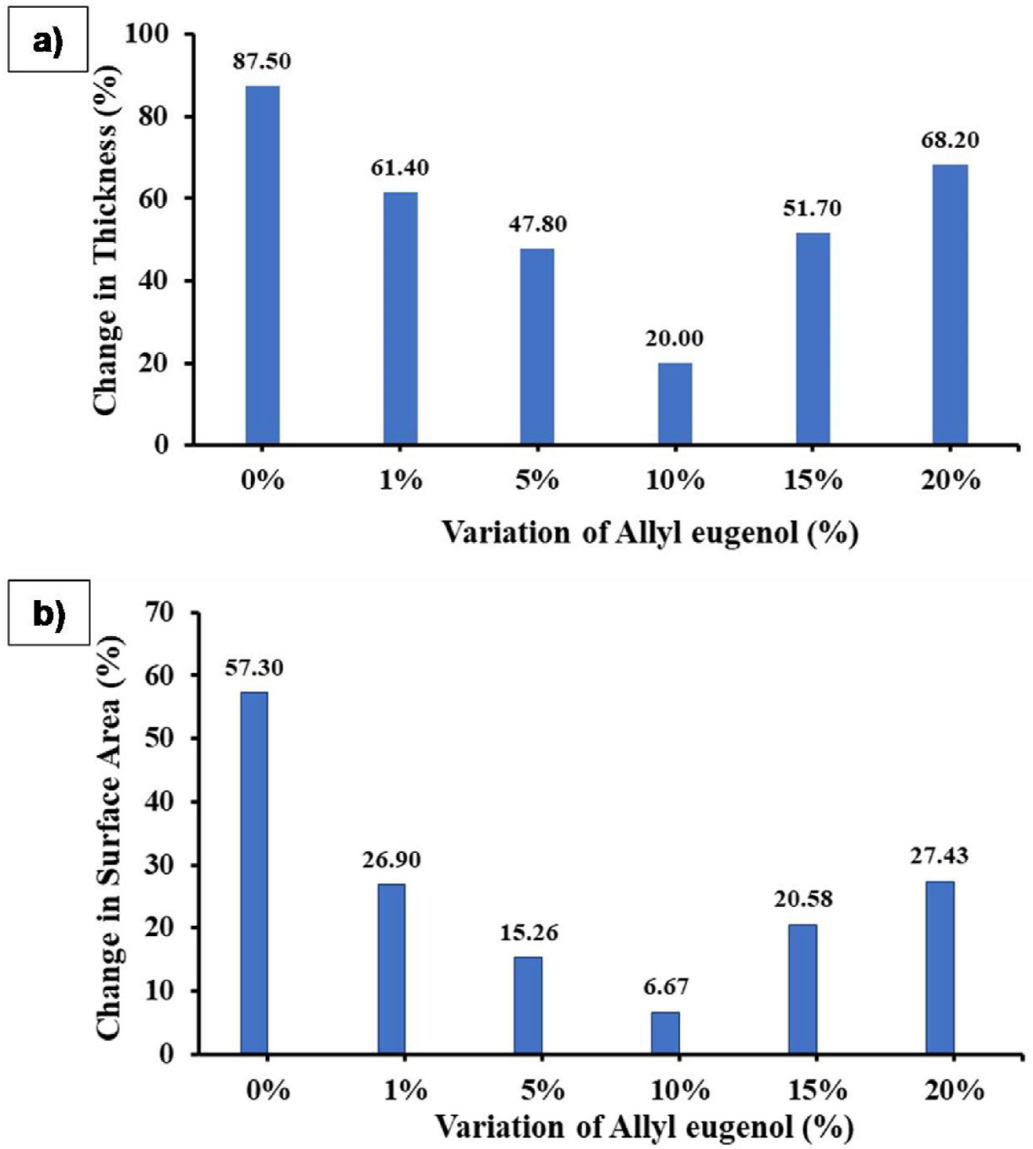
Swelling properties: (a) change in thickness and (b) change in surface area value of the PEAE membrane.

Swelling properties were analyzed from the results of measuring the thickness and surface area of the membrane as presented in Figure 4 (a) and (b). The graph fluctuates with variations in the addition of allyl eugenol. Polyeugenol without allyl eugenol (0% PEAE) experienced the largest change in thickness and surface area at 87.5% and 57.3%, respectively. This value gradually decreased as the PEAE copolymer was formed with variations in the addition of allyl eugenol from 1–20% with minimum conditions or the best conditions at variations of 10% addition.

**Figure 5:**
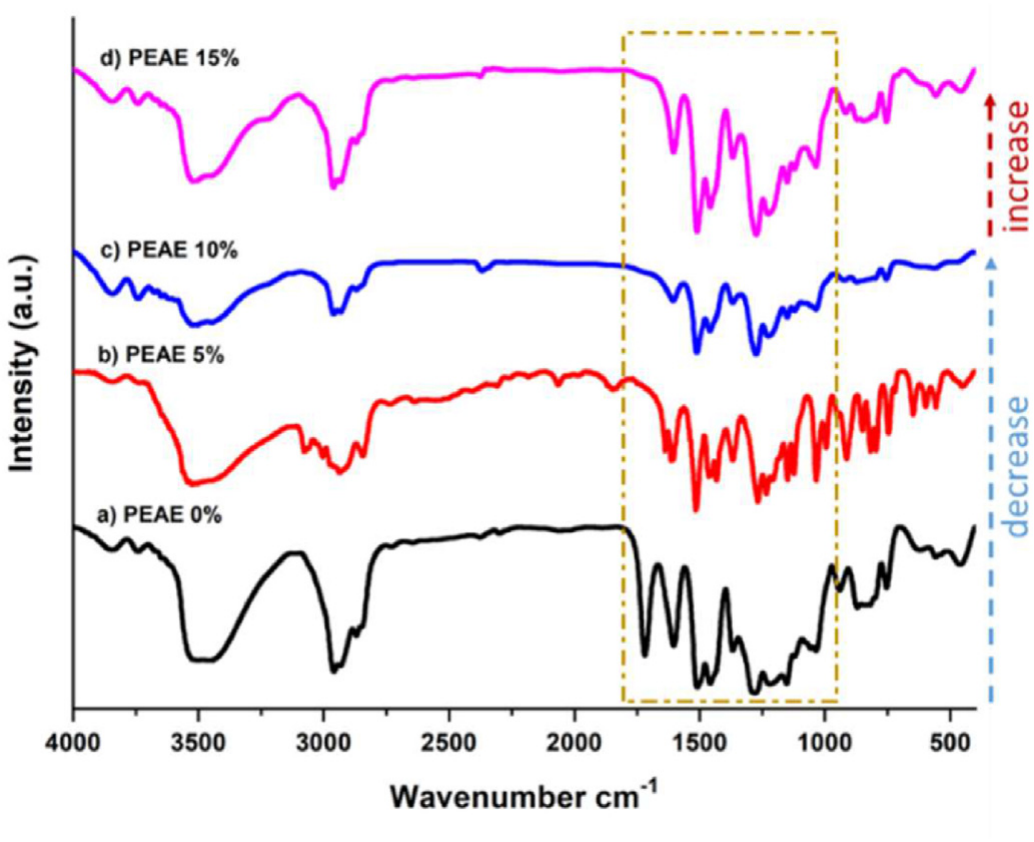
FTIR Spectra (a) polyeugenol (PEAE 0%), (b) PEAE 5%, (c) PEAE 10%, and (d) PEAE 15%.

This indicates that the addition of 1–10% allyl eugenol in the PEAE copolymer forms cross-links resulting in a denser and stronger copolymer and can reduce membrane swelling. While the addition of 15% or more allyl eugenol will increase the swelling again because the greater number of allyl eugenol exceeds the optimum ratio so the bonds between allyl eugenol are getting bigger. This causes the formation of longer branch chains or larger pores, making it easier for water to enter and the swelling to swell again. These results are also supported by the FTIR spectra in Figure 5 where the bond spectral intensity in the fingerprint region of the 0–10% PEAE variation decreased, while the 15% PEAE variation increased the bond intensity.

The higher the swelling property, the easier the polymer will swell. So that it will affect the performance of the membrane on the polymer electrolyte membrane because it is prone to crossover [33]. Swelling of the membrane causes an increase in the diffusion resistance of proton transport across the membrane by increasing the path protons must travel, thereby reducing the performance efficiency of the fuel cell. It can be seen that the lowest degree of swelling was obtained in the PEAE copolymer with a ratio of 10%. Therefore, this PEAE copolymer has the potential to be used as a fuel cell electrolyte polymer.

### 3.2 Synthesis of SPEAE

**Figure 6:**
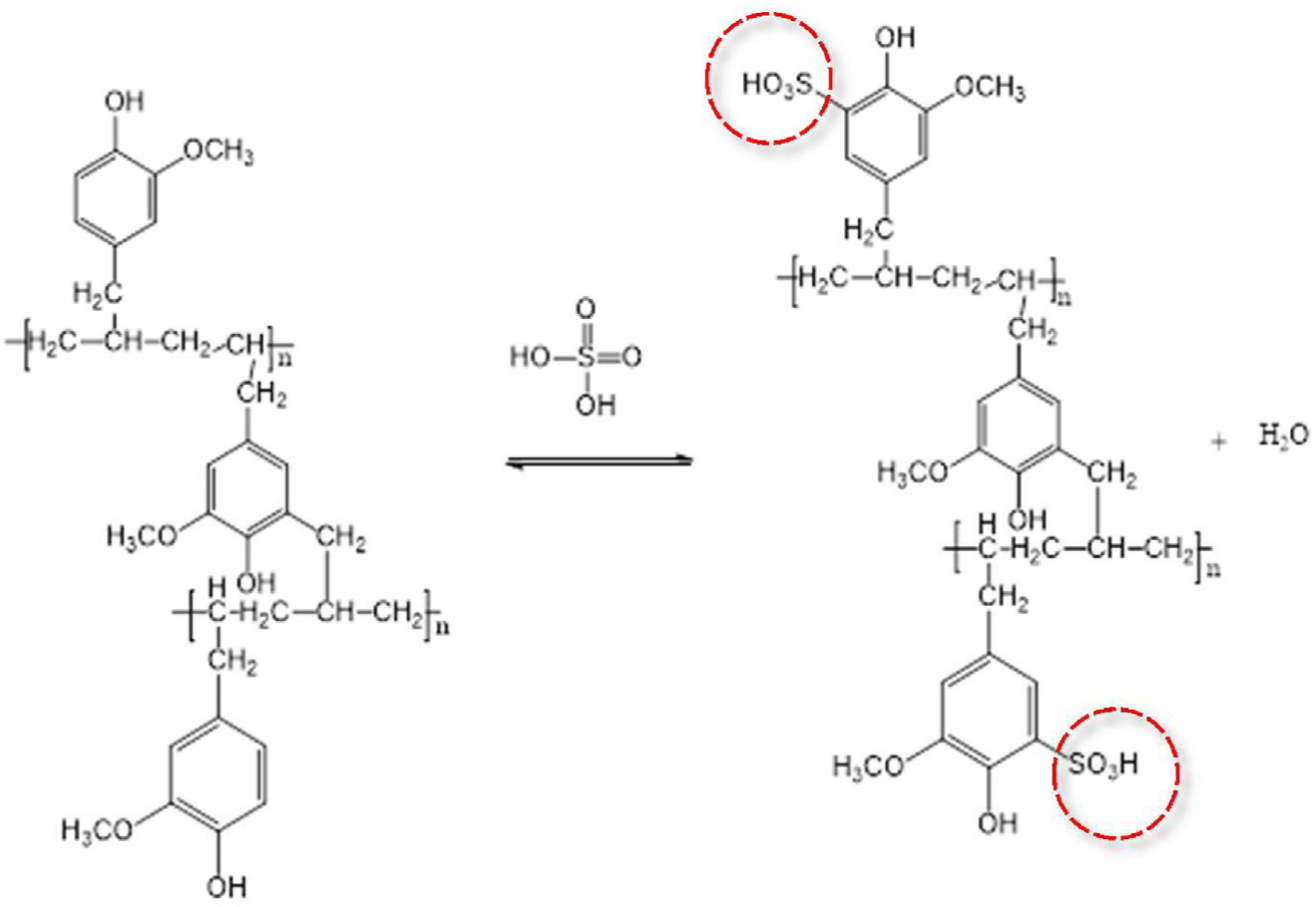
Sulfonation to obtain PEAE sulfonate copolymer.

SPEAE was prepared by sulfonation of PEAE 10%. In this study, sulfonic acid as a sulfonation agent (98%) was used for varying reaction times 1, 2, 3, 4, and 5 h. The difference in sulfonation time aims to determine the optimum sulfonation time used. The yield of sulfonated copolymer results at variations of 1, 2, 3, 4, and 5 h was 91.43; 90.64; 91.32; 89.19, and 90.11%, respectively. An illustration of the PEAE copolymer sulfonation process to SPEAE is shown in Figure 6.

**Figure 7:**
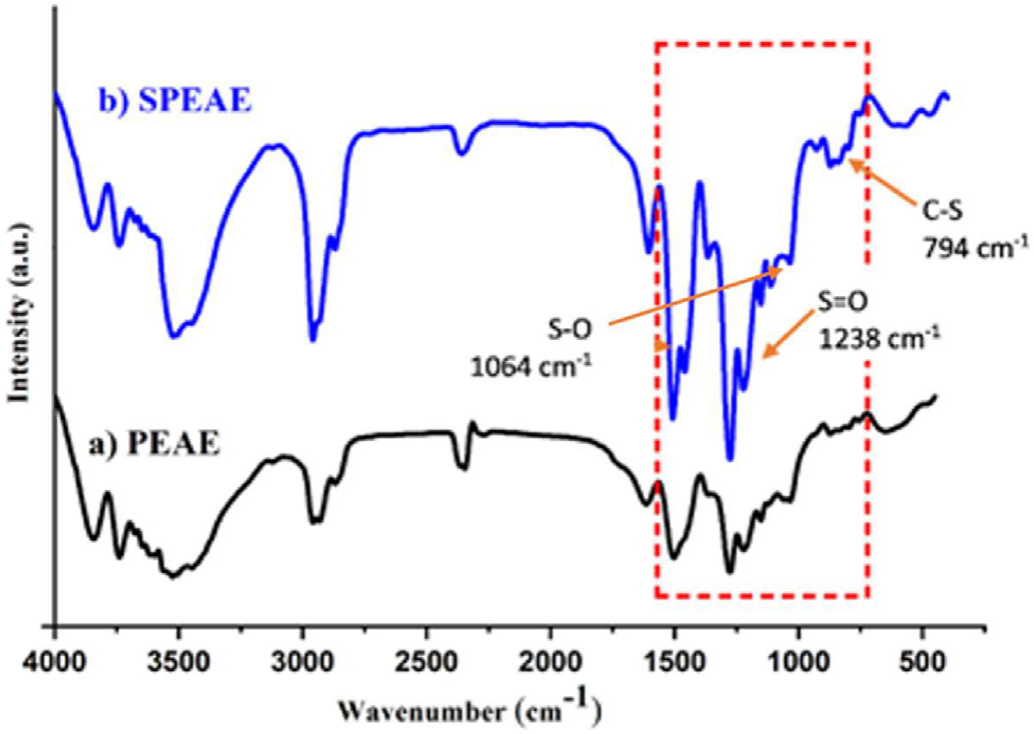
FTIR spectra of (a) PEAE and (b) sulfonated PEAE (SPEAE).

The mechanism of the sulfonation reaction is an electrophilic aromatic substitution reaction involving the SO3 (sulfonic) group of sulfuric acid as an electrophile. The benzene ring acts as an aromatic substrate in the copolymer which will attack the sulfonate group to produce a sulfonated copolymer. The copolymer of PEAE has –OH, –OCH_3_, and –CH_2_ groups which are electron donor groups. This causes the ortho or para position to be more negative so that it is more activated towards electrophilic substitution. The –OH as an electron donor group is stronger than –OCH_3_ and –CH_2_ so the electrophilic substitution will occur at the ortho or para position. The sulfonated copolymers PEAE undergo physical changes in their physical properties, including the change in color to black and the increase in melting point from 84 °C (PEAE copolymer) to 157 °C for sulfonated PEAE copolymer. Characterization for sulfonated PEAE copolymer with an infrared spectrophotometer (FTIR) is shown in Figure 7. While the interpretation of the FTIR spectrum is briefly summarized in Table 1.

**Table 1:** Absorption ranges of PEAE copolymer before and after sulfonation.

Functional group	Absorption range (cm^−1^)	
	Before sulfonation (PEAE)	After sulfonation (SPEAE)
O–H	3523.80	3525.67
C–H alkane	2959.26	2958.28
C=C aromatic	1508.26	1503.10
C–O ether	1223.41	1222.46
S–O	—	1064.14
S=O	—	1238.5
C–S	—	794.3

**Figure 8:**
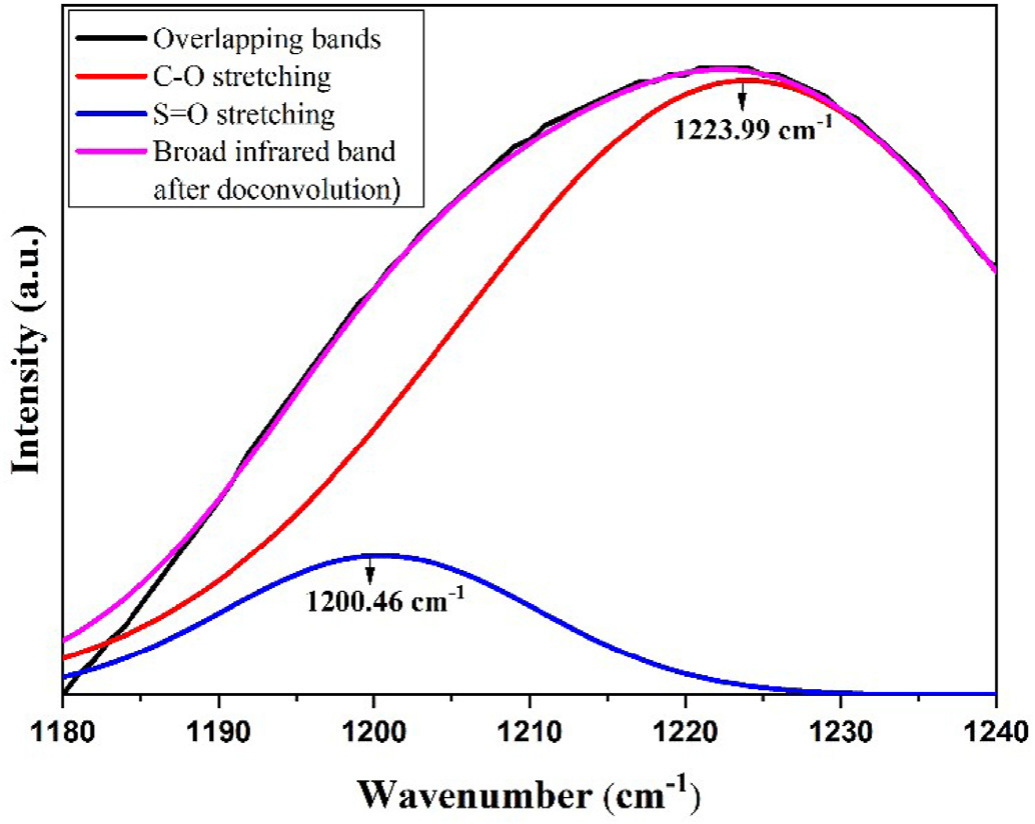
Deconvolution of FTIR spectrum of PEAE sulfonate copolymer from wavenumber of 1180–1250 cm^−1^.

The presence of sulfonate groups is indicated by the appearance of wavenumbers 1064.14, 1238.5, and 794.3 cm^−1^ respectively for vibrations of the S–O, S=O, and C–S groups. S=O vibration at a wavenumber of 1238.5 cm^−1^ is the result of deconvolution because the group overlaps with the C–O ether group in the IR spectrum of the PEAE copolymer after sulfonation. Spectral peak deconvolution was carried out using Fityk software in the range 1180–1250 cm^−1^ as shown in Figure 8. The results showed that there were 4 derived peaks which indicated the absorption of S=O and C–O (ether) groups. Based on some functional group data and deconvolution of FTIR spectra obtained, it can be concluded that the sulfonation reaction has occurred.

**Figure 9:**
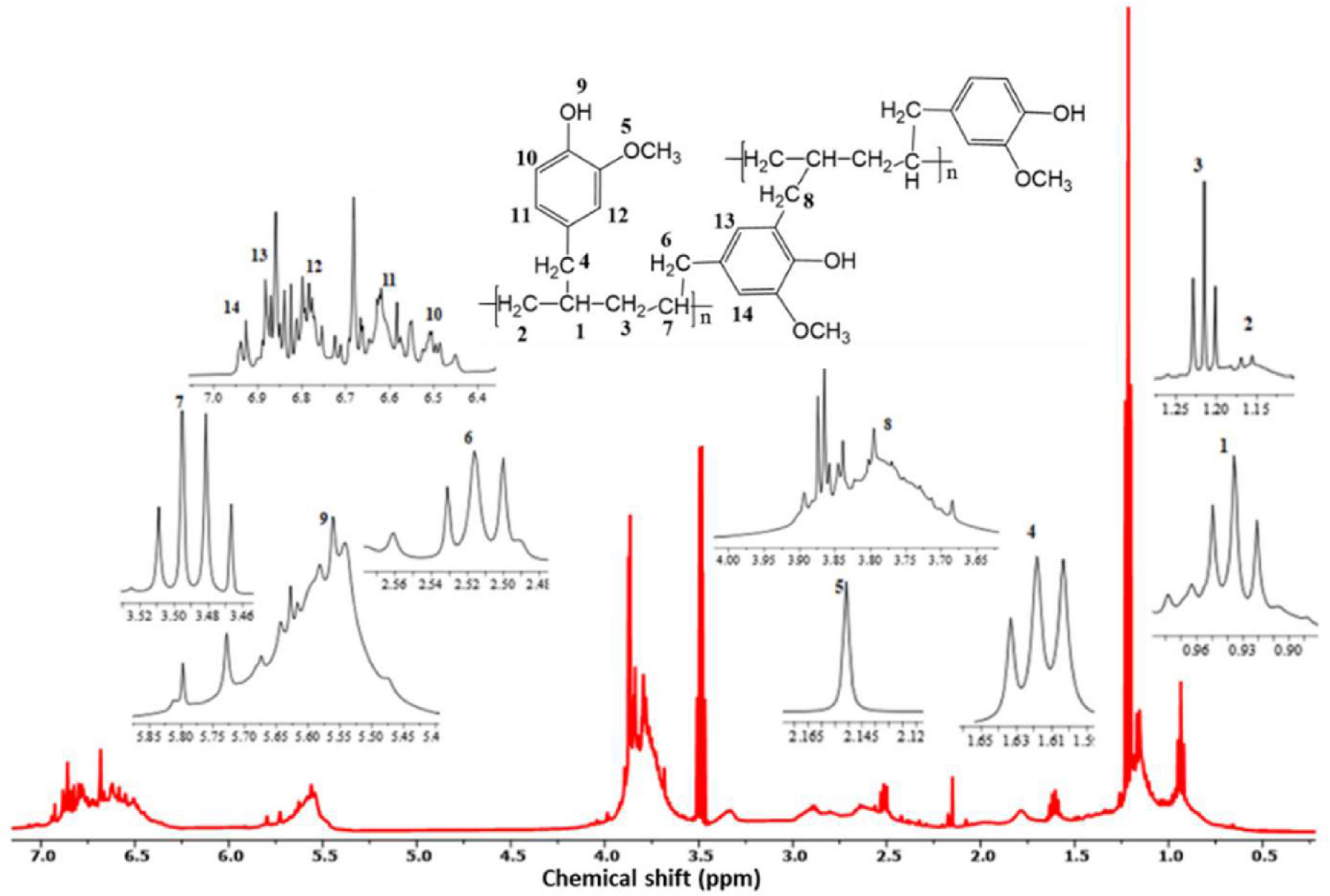
^1^H-NMR PEAE copolymer.

The PEAE membrane was analyzed by ^1^H-NMR spectroscopy to determine the membrane structure of the synthesized copolymer. The ^1^H-NMR spectrum can reveal different types and relative ratios of hydrogen in the polymer structure. The results of the ^1^H-NMR analysis as shown in Figure 9 show that there are 14 types of proton peaks with different environments. The typical proton of PEAE is found in proton 5 which is a typical proton of methoxy and proton 9 which is a typical proton of hydroxy. The peak assignments δ (ppm) are as follows: δH 6.73 (H–Ar), 5.65 (HO-Ph), 3.84 (CH_3_-OPh), 3.5, and 1.23 (aliphatic group).

**Figure 10:**
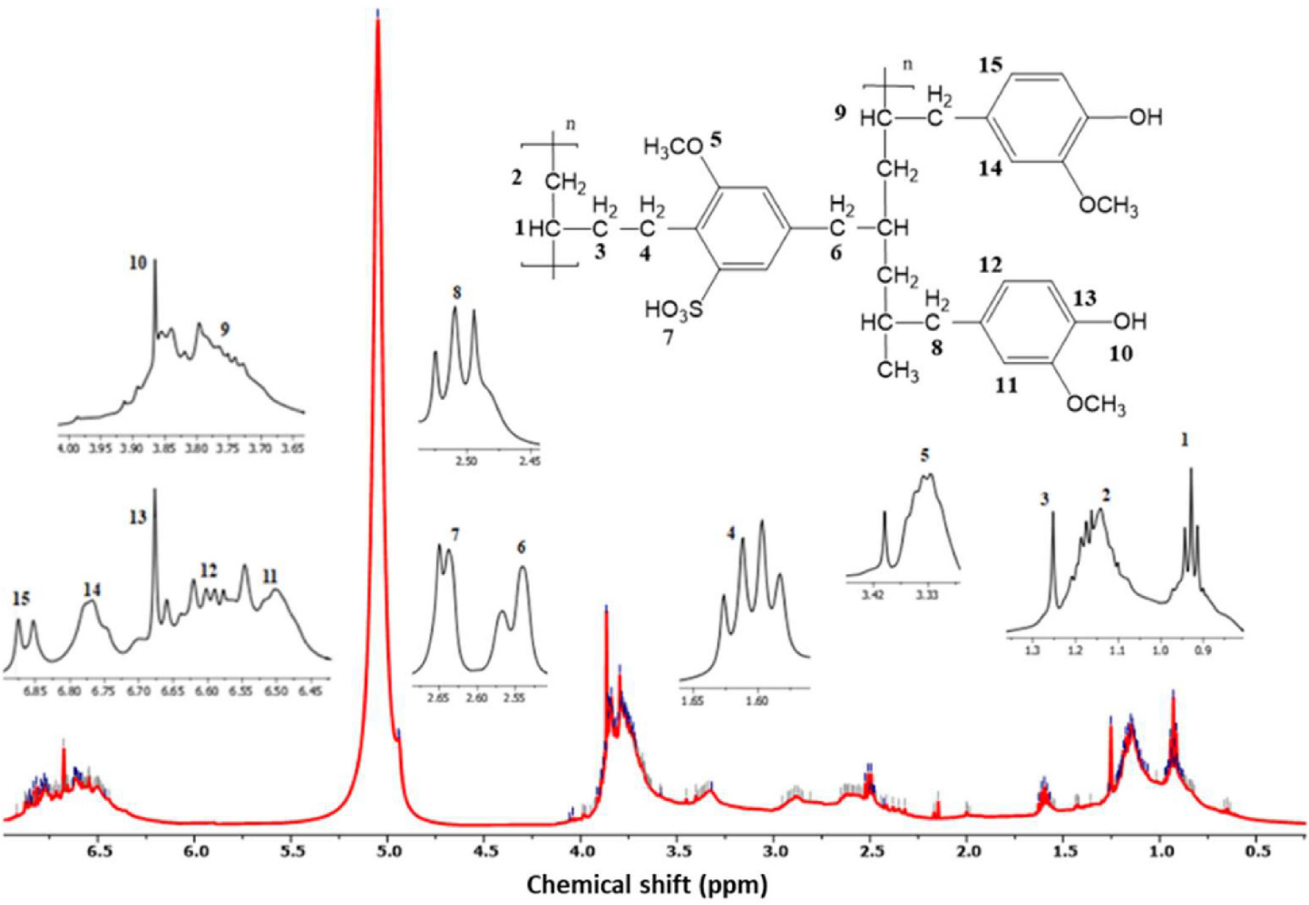
^1^H-NMR sulfonated PEAE copolymer.

The ^1^H-NMR spectrum of the sulfonated PEAE copolymer (SPEAE) structure for 2 h in DMSO-d6 is shown in Figure 10. The peak assignments δ (ppm) are as follows: δH 2.62 (sulfonate group), 6.70 (H–Ar), 5.05 (HO-Ph), 3.80 (CH_3_-OPh), 3.36 and 1.09 (aliphatic group). The typical proton of SPEAE is found in proton 7 which is a typical proton of sulfonate group (SH). This confirms the previous FTIR results in the presence of a sulfonate group in the SPEAE polymer.

**Figure 11:**
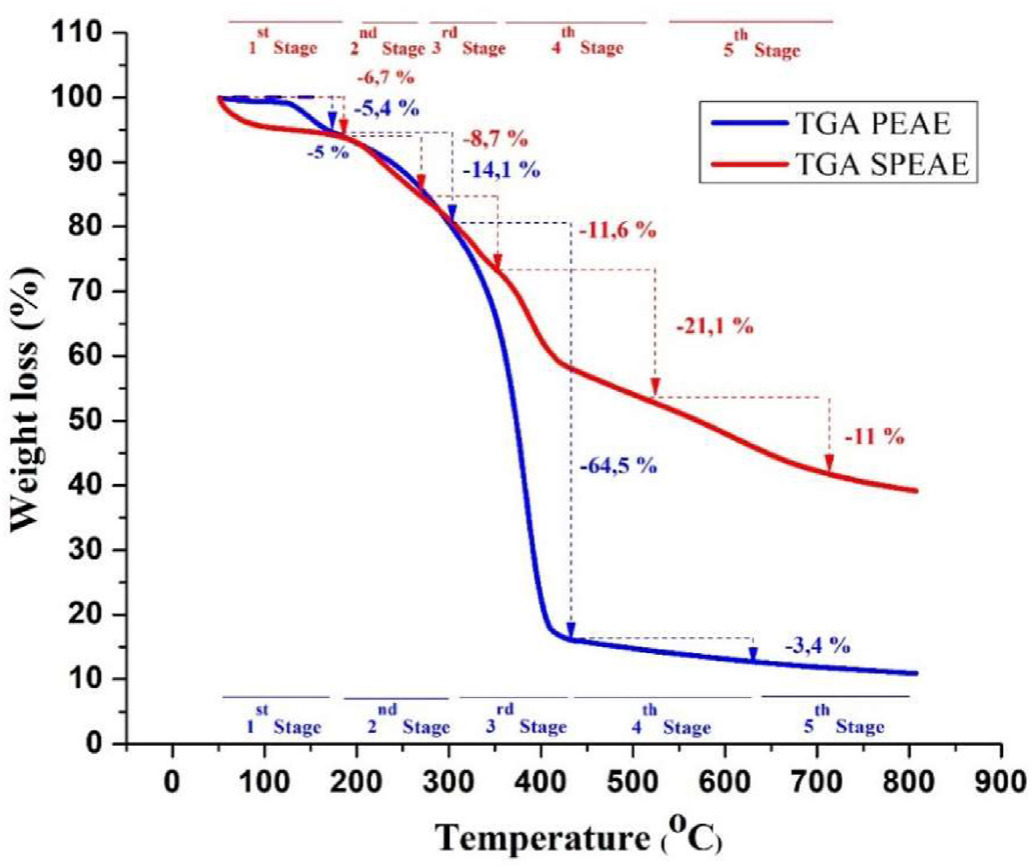
TGA thermogram of PEAE and SPEAE.

Thermogravimetric analysis was performed with TGA-DTG to evaluate whether the sulfonated procedure caused a change in the thermal stability of the copolymer. Figure 11 shows the thermograms for the material before and after sulfonation showing the different degradation steps. TGA-DTG analysis has shown that sulfonated composite membranes (SPEAE) are more stable than pure copolymers (PEAE) and can withstand electrochemical applications at high temperatures. This result is in line with research [40] where sulfonation improves thermal stability.

The first degradation of PEAE and SPEAE were at 126–173.9 °C and 50–196.6 °C, respectively. This mass change is thought to be related to the process of dehydration of the sample, namely the release of water contained in the sample through the evaporation process [41]. The second degradation step observed between 173.9-300 °C due to the initial breaking of the PEAE polymer bond. While in SPEAE the initial breakdown of the sulfonic acid group begins [42]. Furthermore, the third stage in PEAE is the main stage in this analysis which occurs in the temperature range of 300–435 °C by 64.5%. This step is considered the decomposition of the copolymer chain, due to the breaking of the bonds contained in the [41] copolymer. Whereas in SPEAE there was double mass loss associated with the continued destruction and evolution of the sulfonic group around 354 °C by 11.6% and continued degradation of 21.1% until a temperature of 539 °C as the main polymer backbone degradation [41]. In the fourth step of PEAE, the decrease in sample mass was detected in the temperature range of 435–639.5 °C where at this stage the mass loss was 3.4%. This results in the release of volatile substances, which proceed in step 5 leading to the formation of copolymer char products at temperatures above 500 °C. While in SPEAE the decrease in sample mass was detected in the temperature range of 539–740 °C where at this stage the mass loss was 11%. This results in the release of volatile components and leads to the formation of copolymer charcoal products at temperatures above 740 °C.

**Figure 12:**
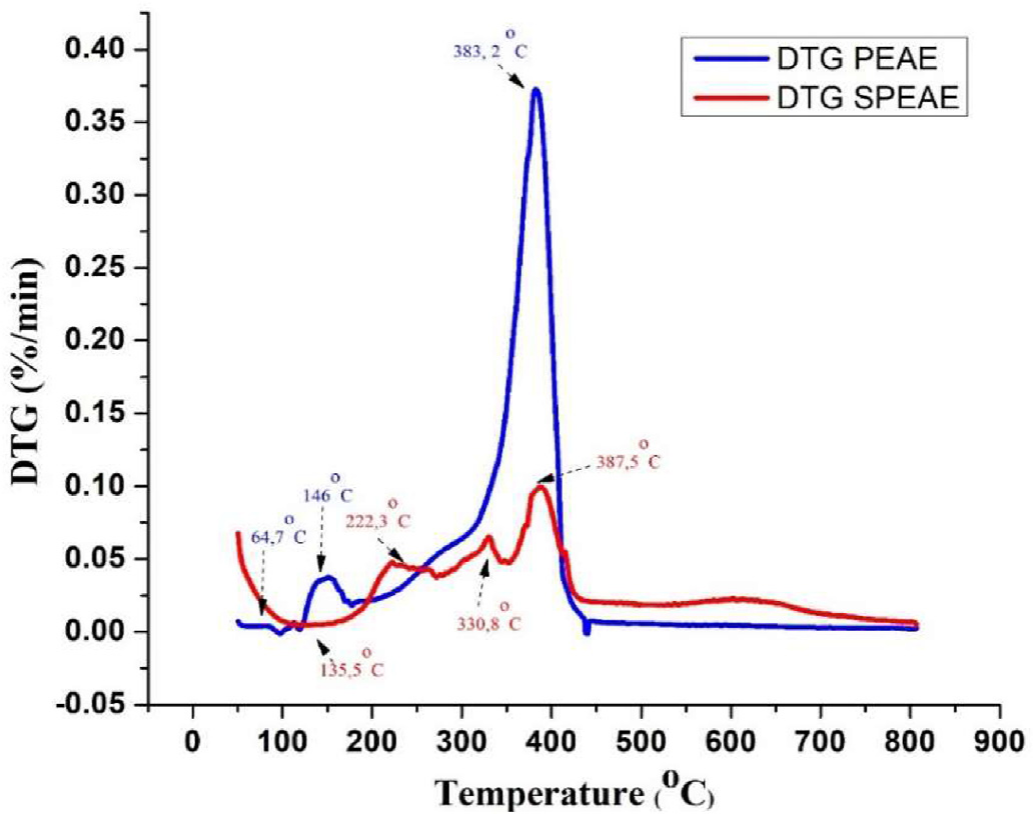
DTG curves of PEAE and SPEAE.

The DTG curve in Figure 12 confirms the TGA results wherein the pure PEAE polymer exhibits a rapid thermal breakdown behavior and a prominent change only due to the degradation of the main polymer backbone in the temperature range of 300–435 °C to be exact at 383.2 °C. On the other hand, the SPEAE polymer matrix showed different stages of degradation in the presence of the sulfonate group degradation process. In addition, the decrease in mass of SPEAE is also lower than that of PEAE which conclusively proves a fairly good thermal stability. The results of the comparison curve of the effect of sulfonation and without sulfonation are in line with those obtained by [41].

**Figure 13:**
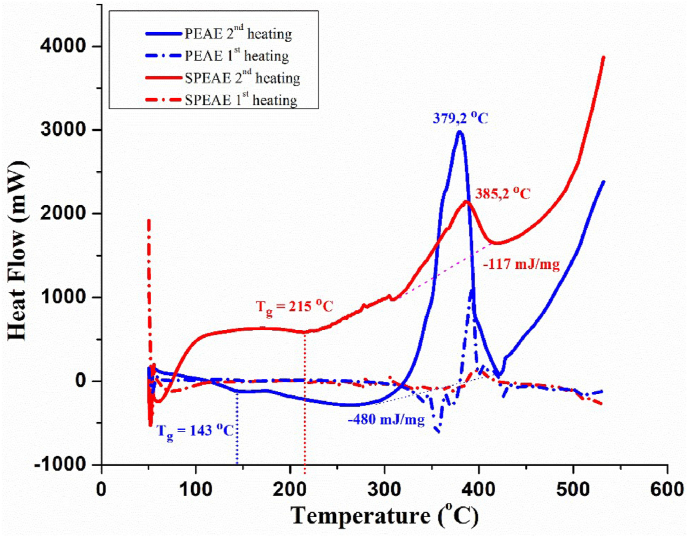
DSC curves of PEAE and SPEAE.

Changes in the copolymer glass transition (T_g_) after synthesis and sulfonation reactions were also evaluated by differential scanning calorimetry (DSC). Figure 13 shows a thermogram according to Thiessen, et al. [43] includes the dehydration area, glass transition temperature, crystallization point, and enthalpy (ΔH). The PEAE and SPEAE DSC curves (2^nd^ heating) show sharp exothermic peaks at 379.2 and 385.2 °C with ΔH −480 mJ/mg and −117 mJ/mg, respectively. This is caused by aromatization and cyclization originating from the degradation of PEAE and SPEAE materials as discussed in the TGA and DTG analysis. This also confirms that PEAE is degraded first and more mass is reduced than SPEAE.

The sulfonation treatment increased the transition temperature (T_g_) of the copolymer membrane from 143 °C to 215 °C. This is due to the influence of the presence of sulfonate groups on the styrene ring. It is known that in the presence of a sulfonic group containing an acid group, it will form hydrogen bonds to the acid group in between chains or in one chain as a backbone. This results in a structure in a more compact direction and increases T_g_ [44]. The Tg temperature depends on the mobility of the polymer molecule bonds, the more difficult it is for a polymer molecule to move, the higher the T_g_ value. Something that inhibits the movement of polymer molecules and the stiffer the main groups of a polymer, the higher its T_g_ value. The attachment of the sulfonate group to the styrene ring can be considered a cause of inhibition of the movement/mobility of the main polymer chain. Sulfonated polystyrene becomes stiff due to cross-linking between chains in polymer [45]. The addition of –SO_3_H groups to the copolymer increases stability by changing the conformation and packing of the chains thereby decreasing the crystallinity and making it more amorphous [46, 47].

#### 3.2.1 Sulfonation degree

**Figure 14:**
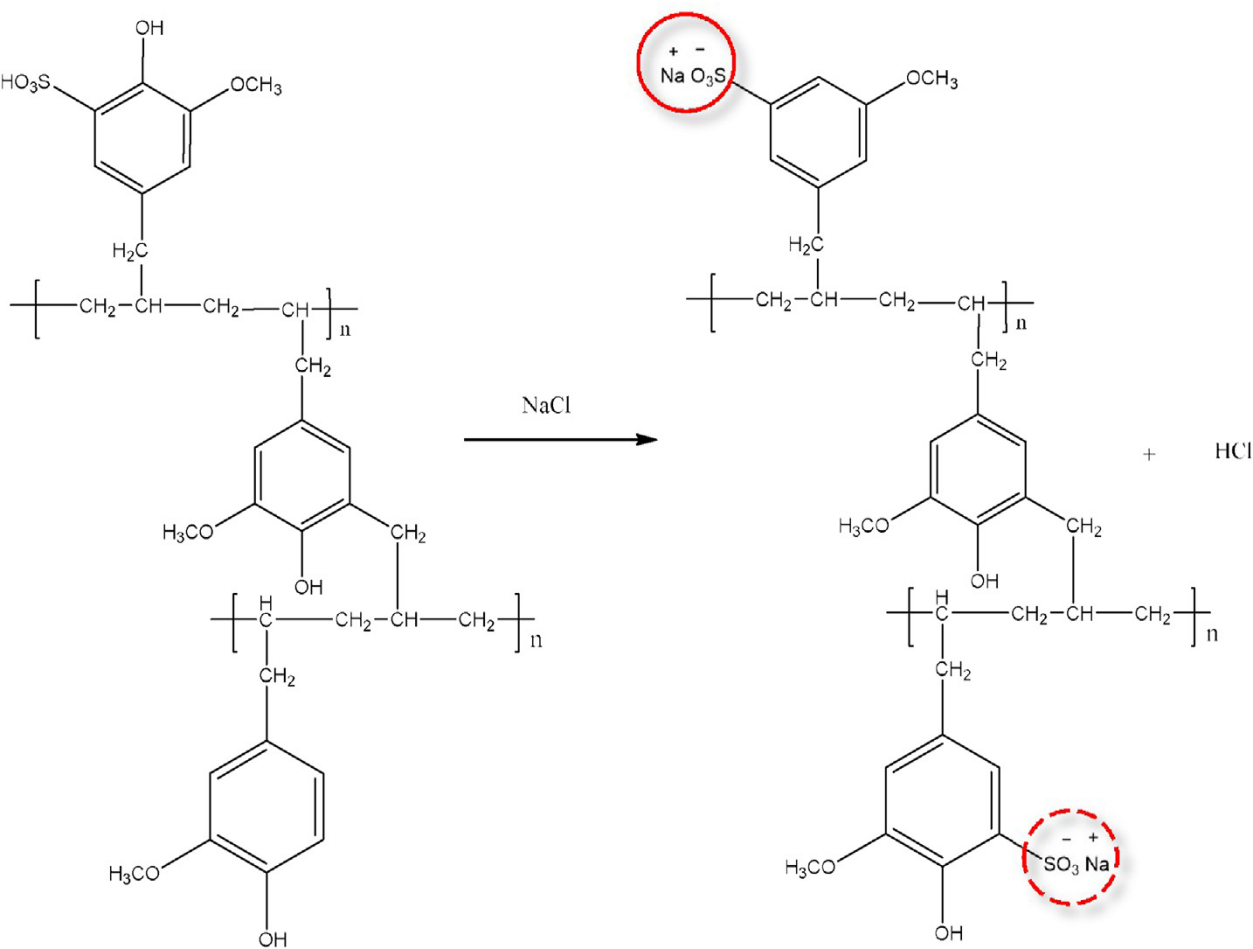
Reaction mechanism measurement of the degree of sulfonation SPEAE.

The measurement of the degree of sulfonation was carried out by the acid-base titration method, namely by immersing the PEAE copolymer in NaCl solution. Soaking in NaCl aims to substitute the H^+^ bound to the SO_3_H group of the sulfonated copolymer with Na^+^. Reaction mechanism measurement of the degree of sulfonation is shown in Figure 14.

**Figure 15:**
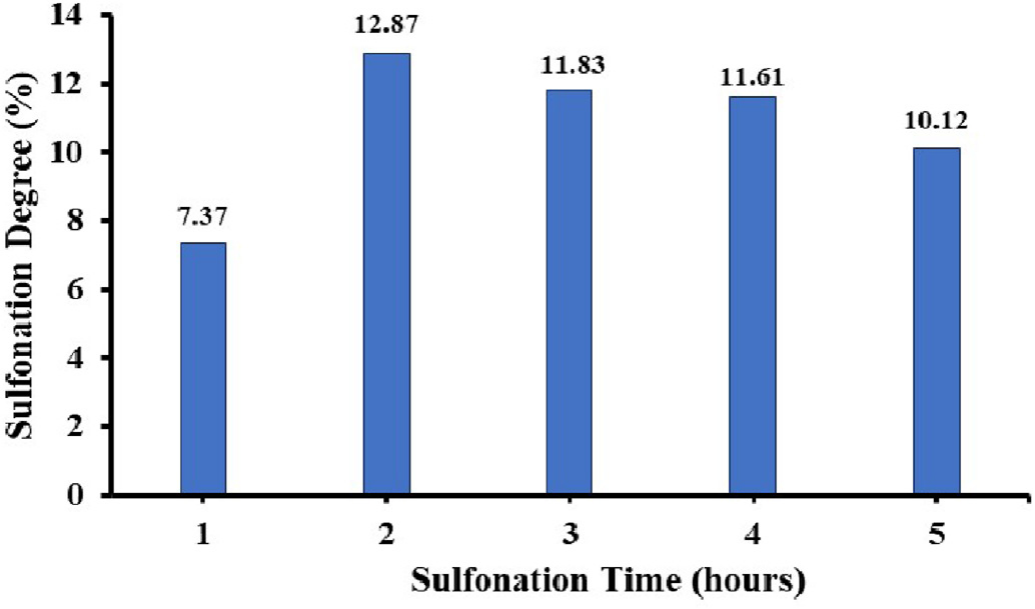
Sulfonation degree of PEAE copolymer with time variation.

The results show that in the first 2 h the degree of sulfonation obtained has increased and after that for the next hour has decreased. This revealed that the sulfonation reaction occurred quickly at the beginning and at the end it become slow. After the sulfonation reaction occurs, the reaction becomes more difficult due to the steric resistance of the –SO_3_H group that has been bonded to the benzene ring. After the –SO_3_H group is substituted into the ring, the rate of electrophilic substitution in the benzene ring will decrease [33]. The longer the sulfonation time, the lower the sulfonation degree due to the reversibility of the sulfonation process. After the reaction proceeds until the number of sulfonate groups attached reaches the optimal limit, the addition of time will cause the release of the sulfonate groups again so that the degree of sulfonation becomes lower. In general, this reaction shows a typical sulfonation reaction tendency where in the early stages the rate is fast and tends to slow down due to the influence of increasing steric resistance of the polymer form. In addition, another factor that may affect the low degree of sulfonation is the presence of water and acids mixed in the sulfonation reaction. This can cause side reactions in the PEAE ester group which is easily hydrolyzed in the presence of sulfuric acid, as shown in Figure 6. The longer the reaction time, the greater the chance of breaking the polymer chain of the ester group, so the shorter polymer chains become easily to be soluble in water. This dissolving causes the sulfonation product obtained to have a small degree of sulfonation and less yield of reaction products. Therefore, it can be concluded that the optimum time for PEAE sulfonation is 2 h as the result of calculating the degree of sulfonation is shown in Figure 15.

#### 3.2.2 Water uptake

**Figure 16:**
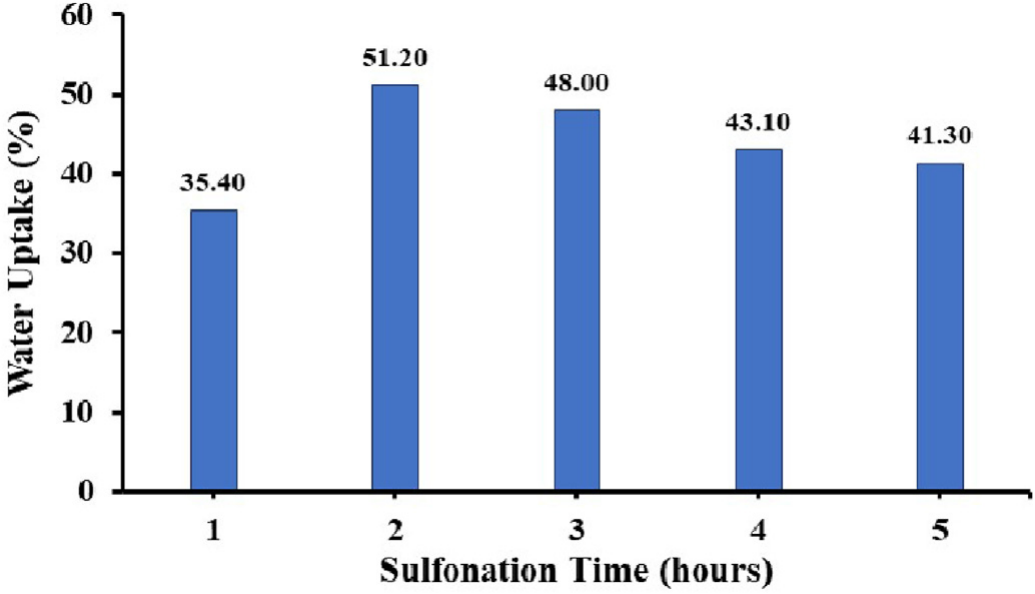
Water uptake of sulfonated PEAE.

The determination of water uptake for each eugenol copolymer and sulfonated allyl eugenol is shown in Figure 16. The water uptake value in the 2 h sulfonated copolymer has increased and the following hour has decreased. This can be shown by increasing the sulfonation degree value, the higher the value for the degree of sulfonation. The higher the –SO_3_H group causes the higher transfer of protons and the value of water uptake.

#### 3.2.3 Cation exchange capacity

This analysis aims to determine the ability of copolymers to exchange cations bound to their functional groups with other or new cations given to the system. The cation exchange capacity is determined by indicating the number of mmol equivalents of sulfonic acid per gram by the weight of the polymer. Analysis of cation exchange capacity (CEC) on the synthesized copolymer is carried out as it will be applied to the fuel cell membrane that requires a polymer material with the ability to exchange ions well.

**Figure 17:**
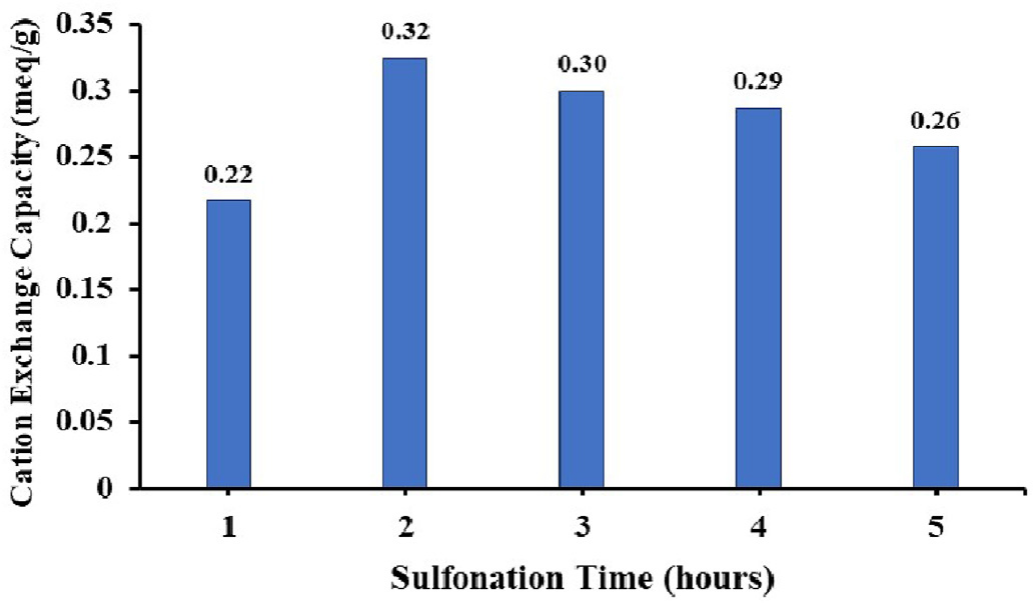
Cation exchange capacity of SPEAE with various sulfonation.

The results of measurements of cation exchange capacity values for PEAE 10% sulfonate copolymers are shown on the graph of the effect of sulfonation time on CEC values in Figure 17.

The largest cation capacity value (0.364 meq/g) is a copolymer with 2 h sulfonation. The sulfonation reaction after 2 h showed a decrease in the CEC value, this could be due to the small number of sulfonate groups that were substituted in the copolymer. The value of cation exchange capacity is proportional to the sulfonation degree value since the sulfonate group (∼SO_3_H) has a role in cation exchange.

#### 3.2.4 Proton conductivity

**Figure 18:**
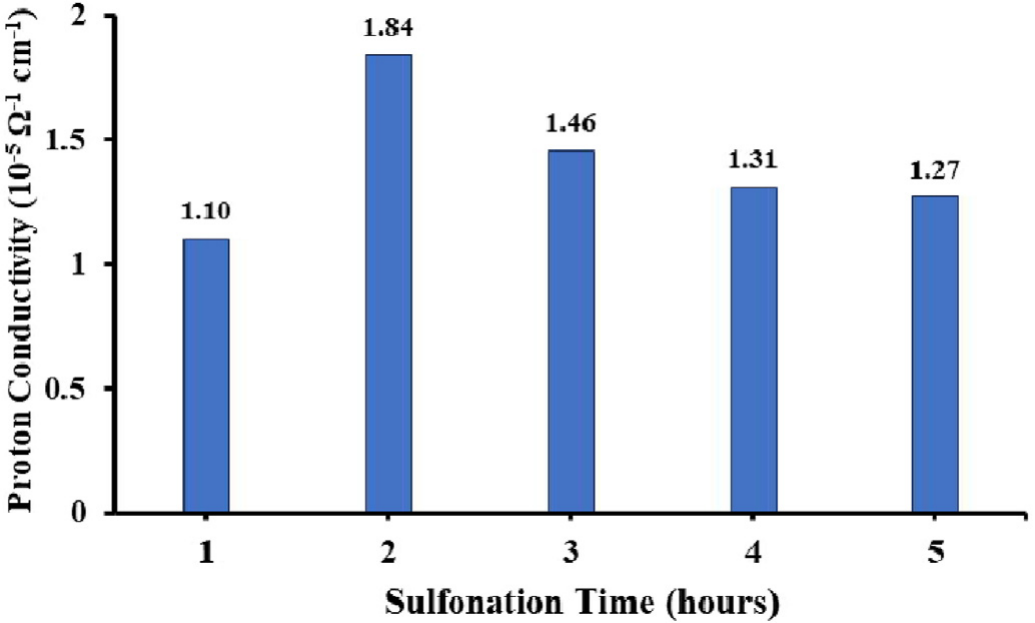
Proton conductivity of sulfonated PEAE with various sulfonation.

Proton conductivity in the copolymer of PEAE sulfonate is measured using an LCR-Meter by measuring the impedance, capacitance, and conductance of the copolymer material. Proton conductivity values are obtained using Eq. (2). The relationship between sulfonation time and proton conductivity can be seen through the graph presented in Figure 18.

The conductivity of pure PEAE copolymer without sulfonation has the lowest proton conductivity value of 0.0314 × 10^−5^ S cm^−1^. This is because the copolymer cannot conduct protons well, the proton contribution is only from the H^+^ phenolic group. Meanwhile, after the sulfonation treatment, there is an additional role of the sulfonate groups in the polymer chain, so that the polymer has a better ability to conduct protons and the value of proton conductivity increases. The sulfonic group, which is this superacid, acts as a proton conductor.

The highest proton conductivity value was obtained in the copolymer of PEAE which was sulfonated in 2 h namely 1.83 × 10^−5^ S cm^−1^. Finally, the proton conductivity decreased at 3, 4, and 5 h during the sulfonation process. This can be explained because the proton conductivity value is related to the DS value, where the greater the DS value, the higher the proton conductivity value. In the results of Figure 17, it is known that the largest DS value is obtained at 2 h of sulfonation. So that the highest conductivity was obtained at 2 h sulfonated SPEAE membrane. This is because the more sulfonic groups that replace the H atom in the PEAE copolymer, the more groups that donate protons (H^+^). The contribution of H^+^ from the hydroxy and sulfonate groups of the PEAE sulfonate copolymer plays a role in proton transfer.

Table 2 summarizes this work with our previous work on eugenol-based polymers reacted with diallyl phthalate [48] and the study carried out by Muller et al [27]. This work is still superior to Muller's work for all polymer properties. Although the sulfonation rate, water uptake, and cation exchange capacity were lower than in our previous studies, the value of the resulting proton conductivity was still higher in this study. With this increase in conductivity, this SPEAE-derived polyeugenol membrane has the potential to be the basis for development as an environmentally friendly membrane in fuel cells using eugenol material.

**Table 2:** Comparison of the polymer properties.

Sample	Sulfonation degree (%)	CEC (meq/g)	Proton conductivity (S.cm^−1^)	Water uptake (%)	Reference
Sulfonated poly(eugenol-co-allyleugenol) (SPEAE)	12.87	0.356	1.83 × 10^−5^	51.2	This work
Previous work [46]	16.55	0.44	8.343 × 10^−6^	73.0	Previous work [46]
(High-impact polystyrene doped with poly (styrene-ethylene-butylene))PS/SEBS and HIPS/SEBS	9–13	0.3–1.24	4.2 × 10^−10^ - 1.5 × 10^−7^	8–36	Muller et al. [27]
Sulfonated poly(ether ether ketone) SPEEK-60	60	1.2	2.7 × 10^−3^	2.5	Liang et al. [49]
sulfonation of poly(ether ether sulfone) SPEES49	37	-	1.72 × 10^−3^	17	Unveren et al. [50]
Nafion 117	-	0.91	2.5 × 10^−3^	19.5	Liang et al. [49]

Although as compared to other sulfonated polymer membranes such as SPEEK-60 by Liang et al. [49], SPEES by Unveren et al [50], and Nafion 117, this study still has a lower proton conductivity. Considering the practical operation of the PEMFC, the current target of DOE (proton conductivity >0.1 S cm−1 at 80 °C and 50% RH) is still challenging. We expect performance improvements in further development as observed for other membrane systems. Further studies to improve the proton conductivity and mechanical properties are being investigated. To further enhance PEM performance, physicochemical tuning and formation of well-connected nanoscale hydrophilic domains in sulfonated aromatic hydrocarbons resembling the structure of Nafion® are essential. These approaches include the use of organic-inorganic composites, thermal annealing, cross-linking, and polymer reinforcement. This combined method is expected to be a new strategy in the design and potential of next-generation PEM materials with high PEMFC performance in the long term. Thus, with further enhancement of the property values of fuel cell membranes, using eugenol-derived biopolymers may replace environmental non-biodegradable fuel cell membranes in the future.

## 4 Conclusion

In this research, the synthesis of a membrane based on sulfonated eugenol-alileugenol copolymer (SPEAE) as a cation exchange membrane was successfully carried out. The copolymer composition of eugenol and allyleugenol with a ratio of 10:1 produces the best mechanical stability with the lowest swelling property value. The variation of sulfonation time with sulfuric acid has an effect on water uptake, cation exchange capacity (CEC), and proton conductivity. The results of the analysis of all the membranes made showed that the best mechanical and thermal properties were obtained from membranes with a sulfonation time of 2 h. SPEAE copolymer under these conditions had yield, degree of sulfonation, water absorption, proton conductivity, and cation exchange capacity of 90.6%, 12.87%, 50.7%, 1.83 × 10^−5^ S cm^−1^, and 0.356 meq/g, respectively. FTIR spectra confirmed the formation of PEAE membranes from the loss of vinyl groups in the polymer while the formation of SPEAE was indicated by the presence of sulfonate groups. The two membranes produced a distinctive peak at ^1^H-NMR which showed a difference in the structure after sulfonation. In addition, the presence of sulfonation also increases the stability of the material in thermal analysis. Further studies to improve proton conductivity and mechanical properties are being investigated in order to increase the potential of eugenol-derived biopolymers as environmentally friendly fuel cell membrane materials in the future.

## 5 Declarations

### Author contribution statement

Ngadiwiyana Ngadiwiyana: Conceived and designed the experiments; Contributed reagents, materials, analysis tools or data; Wrote the paper. Gunawan Gunawan: Performed the experiments; Wrote the paper. Nor Basid Adiwibawa Prasetya: Analyzed and interpreted the data; Wrote the paper. Tutuk D. Kusworo, Heru Susanto: Conceived and designed the experiments.

### Funding statement

This work was supported by Directorate of Research and Community Service of the Ministry of Research and Technology of the Ministry of Education (the 2021 PDUPT research grant with contract number 187–25/UN7.6.1/PP/2021).

### Data availability statement

Data included in article/supplementary material/referenced in article.

### Declaration of interests statement

The authors declare no competing interests.

### Additional information

No additional information is available for this paper.
